# Supplementary material for: Prediction of early breast cancer patient survival using ensembles of hypoxia signatures
Source: PLoS One. 2018 Sep 14;13(9):e0204123. doi: 10.1371/journal.pone.0204123 (PMC6138385; doi:10.1371/journal.pone.0204123)
Supplement: S1 Table — (DOCX) [file pone.0204123.s001.docx]

| Table S1 Overview of preprocessing algorithms. | | | |
| --- | --- | --- | --- |
| Algorithm | Background correction | Normalization | Summarization method |
| RMA | PM probe intensities are corrected array by array using a global model for the distribution of probe intensities. | Quantile normalization is used to impose the same empirical distribution of intensities to each array. | A multi-chip robust model (median polish) is fit to the log2-transformed PM probes for a particular probeset to calculate expression values. |
| GCRMA | Probe sequences are used to calculate the background noise. PM probes are  then corrected with MM probes. | Same as RMA as above. | Similar to RMA as above. |
| MAS5 | Array is divided in K (16) grids. In each  region the 2% lowest probe intensities are used to compute a background value. Each probe is adjusted based upon a weighted average of the backgrounds for each of the regions. Both PM and MM probes are corrected. The PM probes are corrected with MM probes. | Scaling: one array is used as baseline array; other arrays are scaled to have the same mean intensity. | Per array probe values for a particular probeset are combined with the Tukey-Biweight algorithm. |
| MBEI | No background correction is performed. | Invariantset normalization: one array is used as baseline array; arrays are normalized by selecting invariant sets of probes and using these to fit a non-linear relationship between baseline array and other arrays. The non-linear relation is then used to carry out the normalization. | A multi-chip model is fit to each probeset to calculate expression values. |
