# Supplementary material for: Prediction of early breast cancer patient survival using ensembles of hypoxia signatures
Source: PLoS One. 2018 Sep 14;13(9):e0204123. doi: 10.1371/journal.pone.0204123 (PMC6138385; doi:10.1371/journal.pone.0204123)
Supplement: S2 Table — (DOCX) [file pone.0204123.s002.docx]

| Table S2 Summary of 24 preprocessing methods. | | |
| --- | --- | --- |
| Algorithm | Dataset | Annotation |
| RMA | Separate | Default |
| RMA | Separate | Alternative |
| RMA | Merged | Default |
| RMA | Merged | Alternative |
| GCRMA | Separate | Default |
| GCRMA | Separate | Alternative |
| GCRMA | Merged | Default |
| GCRMA | Merged | Alternative |
| MAS5 | Separate | Default |
| MAS5 | Separate | Alternative |
| MAS5 | Merged | Default |
| MAS5 | Merged | Alternative |
| Log_2_-transformed MAS5 | Separate | Default |
| Log_2_-transformed MAS5 | Separate | Alternative |
| Log_2_-transformed MAS5 | Merged | Default |
| Log_2_-transformed MAS5 | Merged | Alternative |
| MBEI | Separate | Default |
| MBEI | Separate | Alternative |
| MBEI | Merged | Default |
| MBEI | Merged | Alternative |
| Log_2_-transformed MBEI | Separate | Default |
| Log_2_-transformed MBEI | Separate | Alternative |
| Log_2_-transformed MBEI | Merged | Default |
| Log_2_-transformed MBEI | Merged | Alternative |
