# Supplementary material for: Prediction of early breast cancer patient survival using ensembles of hypoxia signatures
Source: PLoS One. 2018 Sep 14;13(9):e0204123. doi: 10.1371/journal.pone.0204123 (PMC6138385; doi:10.1371/journal.pone.0204123)
Supplement: S3 Table — (DOCX) [file pone.0204123.s003.docx]

| Table S3 Overview of hypoxia prognostic signatures. | | | |
| --- | --- | --- | --- |
| Signature | No. of genes | Study description | Citation |
| Buffa metagene | 51 | Omnibus searched for gene expression studies/data sets in cancer, in which 8 were selected. Notably, Winter *et al.* was used as a training data set.  Tissue: frozen material extracted prior to receipt of chemotherapy, radiotherapy or adjuvant treatment  Microarray platform: Affymetrix U133A, B and plus2  Normalization method: GCRMA | [[1](#_ENREF_1)] |
| Winter metagene | 101 | Tissue: surgical specimens of untreated head and neck squamous cell carcinomas  Microarray platform: Affymetrix U133plus2 GeneChips  Normalization method: GCRMA | [[2](#_ENREF_2)] |
| Hu | 13 | Tissue: breast tumor  Microarray: Agilent Human oligonucleotide microarrays scanned on an Axon GenePix 4000B  Normalization method: Lowess | [[3](#_ENREF_3)] |
| Sorensen | 28 | Cell line: human uterine cervix squamous cell carcinoma  Hypoxic conditions: 5%, 1%, 0.1%, 0.01% and 0% oxygen,  Microarray: Human Genome U133 Plus 2.0 Array Normalization: MAS 5.0 | [[4](#_ENREF_4)] |

1. Buffa FM, Harris AL, West CM, Miller CJ: **Large meta-analysis of multiple cancers reveals a common, compact and highly prognostic hypoxia metagene**. *British journal of cancer* 2010, **102**(2):428-435.

2. Winter SC, Buffa FM, Silva P, Miller C, Valentine HR, Turley H, Shah KA, Cox GJ, Corbridge RJ, Homer JJ *et al*: **Relation of a hypoxia metagene derived from head and neck cancer to prognosis of multiple cancers**. *Cancer research* 2007, **67**(7):3441-3449.

3. Hu Z, Fan C, Livasy C, He X, Oh DS, Ewend MG, Carey LA, Subramanian S, West R, Ikpatt F *et al*: **A compact VEGF signature associated with distant metastases and poor outcomes**. *BMC medicine* 2009, **7**:9.

4. Sorensen BS, Toustrup K, Horsman MR, Overgaard J, Alsner J: **Identifying pH independent hypoxia induced genes in human squamous cell carcinomas in vitro**. *Acta oncologica* 2010, **49**(7):895-905.
