# Supplementary material for: Prediction of early breast cancer patient survival using ensembles of hypoxia signatures
Source: PLoS One. 2018 Sep 14;13(9):e0204123. doi: 10.1371/journal.pone.0204123 (PMC6138385; doi:10.1371/journal.pone.0204123)
Supplement: S4 Table — (DOCX) [file pone.0204123.s004.docx]

| Table S4 Summary of votes used to calculate engineered variables. | | | | | | | | | | | | | |
| --- | --- | --- | --- | --- | --- | --- | --- | --- | --- | --- | --- | --- | --- |
| Algorithm | Total Votes | Total votes separate | Total votes merged | Total votes RMA | Total votes GCRMA | Total votes MBEI | Total votes MAS5 | Total votes RMA | Total votes MBEI log_2_ | Total votes MAS5 log_2_ | Total votes RMA and MAS5 | Total votes default | Total votes alternative |
| Separate RMA default | ✓ | ✓ |  | ✓ |  |  |  | ✓ |  |  | ✓ | ✓ |  |
| Separate RMA alternative | ✓ | ✓ |  | ✓ |  |  |  | ✓ |  |  | ✓ |  | ✓ |
| Merged RMA default | ✓ |  | ✓ | ✓ |  |  |  | ✓ |  |  | ✓ | ✓ |  |
| Merged RMA alternative | ✓ |  | ✓ | ✓ |  |  |  | ✓ |  |  | ✓ |  | ✓ |
| Separate GCRMA default | ✓ | ✓ |  |  | ✓ |  |  |  |  |  |  | ✓ |  |
| Separate GCRMA alternative | ✓ | ✓ |  |  | ✓ |  |  |  |  |  |  |  | ✓ |
| Merged GCRMA default | ✓ |  | ✓ |  | ✓ |  |  |  |  |  |  | ✓ |  |
| Merged GCRMA alterntive | ✓ |  | ✓ |  | ✓ |  |  |  |  |  |  |  | ✓ |
| Separate MAS5 default log_2_ | ✓ | ✓ |  |  |  | ✓ |  |  |  | ✓ | ✓ | ✓ |  |
| Separate MAS5 alternative log_2_ | ✓ | ✓ |  |  |  | ✓ |  |  |  | ✓ | ✓ |  | ✓ |
| Merged MAS5 default log_2_ | ✓ |  | ✓ |  |  | ✓ |  |  |  | ✓ | ✓ | ✓ |  |
| Merged MAS5 alternative log_2_ | ✓ |  | ✓ |  |  | ✓ |  |  |  | ✓ | ✓ |  | ✓ |
| Separate MAS5 default log_2_ | ✓ | ✓ |  |  |  | ✓ |  |  |  |  | ✓ | ✓ |  |
| Separate MAS5 alternative | ✓ | ✓ |  |  |  | ✓ |  |  |  |  | ✓ |  | ✓ |
| Merged MAS5 default | ✓ |  | ✓ |  |  | ✓ |  |  |  |  | ✓ | ✓ |  |
| Merged MAS5 alternative | ✓ |  | ✓ |  |  | ✓ |  |  |  |  | ✓ |  | ✓ |
| Separate MBEI default log_2_ | ✓ | ✓ |  |  |  |  | ✓ |  | ✓ |  |  | ✓ |  |
| Separate MBEI alternative log_2_ | ✓ | ✓ |  |  |  |  | ✓ |  | ✓ |  |  |  | ✓ |
| Merged MBEI default log_2_ | ✓ |  | ✓ |  |  |  | ✓ |  | ✓ |  |  | ✓ |  |
| Merged MBEI alternative log_2_ | ✓ |  | ✓ |  |  |  | ✓ |  | ✓ |  |  |  | ✓ |
| Separate MBEI default | ✓ | ✓ |  |  |  |  | ✓ |  |  |  |  | ✓ |  |
| Separate MBEI alternative | ✓ | ✓ |  |  |  |  | ✓ |  |  |  |  |  | ✓ |
| Merged MBEI default | ✓ |  | ✓ |  |  |  | ✓ |  |  |  |  | ✓ |  |
| Merged MBEI alternative | ✓ |  | ✓ |  |  |  | ✓ |  |  |  |  |  | ✓ |
