# Supplementary material for: Prediction of early breast cancer patient survival using ensembles of hypoxia signatures
Source: PLoS One. 2018 Sep 14;13(9):e0204123. doi: 10.1371/journal.pone.0204123 (PMC6138385; doi:10.1371/journal.pone.0204123)
Supplement: S5 Table — (DOCX) [file pone.0204123.s005.docx]

| Table S5 Hazard ratios and 95% confidence intervals obtained for each of the 24 preprocessing methods, the random forest classifiers evaluated, and the simple unanimous vote classifier, per signature (HG-U133A microarray platform). | | | | | | |
| --- | --- | --- | --- | --- | --- | --- |
| Classifier | | **Signature** | **HR** | ***P*-value** | **Upper 95% CI** | **Lower 95% CI** |
| Preprocessing pipeline | |  |  |  |  |  |
|  | single_RMA_default_group | Buffa | 2.09 | 1.4E-11 | 2.59 | 1.69 |
|  | single_MAS5_default_group | Buffa | 1.92 | 1.4E-09 | 2.38 | 1.56 |
|  | single_MBEI_default_group | Buffa | 2.11 | 9.3E-12 | 2.61 | 1.7 |
|  | single_GCRMA_default_group | Buffa | 2.04 | 4.8E-11 | 2.53 | 1.65 |
|  | single_MAS5_log2_default_group | Buffa | 1.92 | 1.4E-09 | 2.38 | 1.56 |
|  | single_MBEI_log2_default_group | Buffa | 2.11 | 9.3E-12 | 2.61 | 1.7 |
|  | single_RMA_alternative_group | Buffa | 1.98 | 2.0E-10 | 2.44 | 1.6 |
|  | single_MAS5_alternative_group | Buffa | 2.16 | 1.0E-12 | 2.67 | 1.75 |
|  | single_MBEI_alternative_group | Buffa | 2.09 | 9.7E-12 | 2.58 | 1.69 |
|  | single_GCRMA_alternative_group | Buffa | 1.92 | 1.2E-09 | 2.36 | 1.55 |
|  | single_MAS5_log2_alternative_group | Buffa | 2.16 | 1.0E-12 | 2.67 | 1.75 |
|  | single_MBEI_log2_alternative_group | Buffa | 2.09 | 9.7E-12 | 2.58 | 1.69 |
|  | all_GCRMA_default_group | Buffa | 1.97 | 3.6E-10 | 2.44 | 1.59 |
|  | all_MAS5_log2_default_group | Buffa | 1.9 | 2.8E-09 | 2.34 | 1.54 |
|  | all_MBEI_log2_default_group | Buffa | 2.24 | 2.2E-13 | 2.78 | 1.81 |
|  | all_GCRMA_alternative_group | Buffa | 2 | 1.2E-10 | 2.46 | 1.62 |
|  | all_MAS5_log2_alternative_group | Buffa | 1.85 | 8.1E-09 | 2.29 | 1.5 |
|  | all_MBEI_log2_alternative_group | Buffa | 2.05 | 2.7E-11 | 2.53 | 1.66 |
|  | all_RMA_default_group | Buffa | 2.04 | 6.1E-11 | 2.52 | 1.65 |
|  | all_MAS5_default_group | Buffa | 1.9 | 2.8E-09 | 2.34 | 1.54 |
|  | all_RMA_alternative_group | Buffa | 1.97 | 2.3E-10 | 2.43 | 1.6 |
|  | all_MAS5_alternative_group | Buffa | 1.85 | 8.1E-09 | 2.29 | 1.5 |
|  | all_MBEI_default_group | Buffa | 2.24 | 2.2E-13 | 2.78 | 1.81 |
|  | all_MBEI_alternative_group | Buffa | 2.05 | 2.7E-11 | 2.53 | 1.66 |
| Unanimous classifier | |  |  |  |  |  |
|  | unanimous | Buffa | 2.92 | 9.9E-14 | 3.88 | 2.2 |
| Random forest classifiers | |  |  |  |  |  |
|  | preprocessing ensemble (unanimous patients only) | Buffa | 1.32 | 1.2E-01 | 1.88 | 0.93 |
|  | preprocessing ensemble (all patients) | Buffa | 2.09 | 3.0E-12 | 2.58 | 1.7 |
|  | engineered variables (unanimous patients only) | Buffa | 0.99 | 9.8E-01 | 1.44 | 0.69 |
|  | engineered variables (all patients) | Buffa | 2.15 | 4.1E-13 | 2.64 | 1.75 |
|  | preprocessing and engineered variables (all patients) | Buffa | 1.97 | 1.1E-10 | 2.42 | 1.6 |
|  | Boruta algorithm selected features | Buffa | 2.01 | 3.6E-11 | 2.46 | 1.63 |
| Preprocessing pipeline | |  |  |  |  |  |
|  | single_RMA_default_group | Winter | 1.87 | 6.6E-09 | 2.3 | 1.51 |
|  | single_MAS5_default_group | Winter | 1.76 | 1.4E-07 | 2.16 | 1.42 |
|  | single_MBEI_default_group | Winter | 1.79 | 5.8E-08 | 2.21 | 1.45 |
|  | single_GCRMA_default_group | Winter | 2.22 | 4.0E-13 | 2.76 | 1.79 |
|  | single_MAS5_log2_default_group | Winter | 1.76 | 1.4E-07 | 2.16 | 1.42 |
|  | single_MBEI_log2_default_group | Winter | 1.79 | 5.8E-08 | 2.21 | 1.45 |
|  | single_RMA_alternative_group | Winter | 2.01 | 8.1E-11 | 2.48 | 1.63 |
|  | single_MAS5_alternative_group | Winter | 1.76 | 8.7E-08 | 2.16 | 1.43 |
|  | single_MBEI_alternative_group | Winter | 1.92 | 7.6E-10 | 2.37 | 1.56 |
|  | single_GCRMA_alternative_group | Winter | 2.22 | 2.0E-13 | 2.75 | 1.8 |
|  | single_MAS5_log2_alternative_group | Winter | 1.76 | 8.7E-08 | 2.16 | 1.43 |
|  | single_MBEI_log2_alternative_group | Winter | 1.92 | 7.6E-10 | 2.37 | 1.56 |
|  | all_RMA_default_group | Winter | 2.07 | 2.4E-11 | 2.56 | 1.67 |
|  | all_MAS5_default_group | Winter | 1.88 | 5.2E-09 | 2.32 | 1.52 |
|  | all_MBEI_default_group | Winter | 2 | 1.6E-10 | 2.48 | 1.62 |
|  | all_GCRMA_default_group | Winter | 2.13 | 4.4E-12 | 2.64 | 1.72 |
|  | all_MAS5_log2_default_group | Winter | 1.88 | 5.2E-09 | 2.32 | 1.52 |
|  | all_MBEI_log2_default_group | Winter | 2 | 1.6E-10 | 2.48 | 1.62 |
|  | all_RMA_alternative_group | Winter | 2 | 1.7E-10 | 2.47 | 1.62 |
|  | all_MAS5_alternative_group | Winter | 1.93 | 8.1E-10 | 2.38 | 1.56 |
|  | all_MBEI_alternative_group | Winter | 1.97 | 2.6E-10 | 2.43 | 1.6 |
|  | all_GCRMA_alternative_group | Winter | 2.07 | 8.5E-12 | 2.56 | 1.68 |
|  | all_MAS5_log2_alternative_group | Winter | 1.93 | 8.1E-10 | 2.38 | 1.56 |
|  | all_MBEI_log2_alternative_group | Winter | 1.97 | 2.6E-10 | 2.43 | 1.6 |
| Unanimous classifier | |  |  |  |  |  |
|  | unanimous | Winter | 3.48 | 5.0E-12 | 4.95 | 2.44 |
| Random forest classifiers | |  |  |  |  |  |
|  | preprocessing ensemble (unanimous patients only) | Winter | 1.5 | 4.6E-03 | 1.98 | 1.13 |
|  | preprocessing ensemble (all patients) | Winter | 2.24 | 1.4E-14 | 2.75 | 1.82 |
|  | engineered variables (unanimous patients only) | Winter | 1.39 | 2.7E-02 | 1.85 | 1.04 |
|  | engineered variables (all patinets) | Winter | 2.39 | 9.9E-17 | 2.93 | 1.94 |
|  | preprocessing and engineered variables (all patients) | Winter | 2.25 | 9.6E-15 | 2.76 | 1.83 |
|  | Boruta algorithm selected features | Winter | 2.12 | 7.4E-13 | 2.6 | 1.72 |
| Preprocessing pipeline | |  |  |  |  |  |
|  | single_RMA_default_group | Hu | 1.32 | 8.3E-03 | 1.62 | 1.07 |
|  | single_MAS5_default_group | Hu | 1.32 | 7.5E-03 | 1.62 | 1.08 |
|  | single_GCRMA_default_group | Hu | 1.37 | 2.8E-03 | 1.68 | 1.11 |
|  | single_MBEI_default_group | Hu | 1.25 | 3.5E-02 | 1.53 | 1.02 |
|  | single_RMA_alternative_group | Hu | 1.65 | 1.4E-06 | 2.02 | 1.35 |
|  | single_MAS5_alternative_group | Hu | 1.43 | 6.2E-04 | 1.75 | 1.16 |
|  | single_GCRMA_alternative_group | Hu | 1.66 | 1.2E-06 | 2.03 | 1.35 |
|  | single_MBEI_alternative_group | Hu | 1.63 | 2.2E-06 | 2 | 1.33 |
|  | all_RMA_default_group | Hu | 1.34 | 5.0E-03 | 1.65 | 1.09 |
|  | all_MAS5_default_group | Hu | 1.33 | 7.0E-03 | 1.63 | 1.08 |
|  | all_RMA_alternative_group | Hu | 1.67 | 8.9E-07 | 2.04 | 1.36 |
|  | all_MAS5_alternative_group | Hu | 1.49 | 1.3E-04 | 1.82 | 1.21 |
|  | all_MBEI_default_group | Hu | 1.38 | 1.9E-03 | 1.7 | 1.13 |
|  | all_MBEI_alternative_group | Hu | 1.65 | 1.7E-06 | 2.02 | 1.34 |
|  | single_MAS5_log2_default_group | Hu | 1.32 | 7.5E-03 | 1.62 | 1.08 |
|  | single_MBEI_log2_default_group | Hu | 1.25 | 3.5E-02 | 1.53 | 1.02 |
|  | single_MAS5_log2_alternative_group | Hu | 1.43 | 6.2E-04 | 1.75 | 1.16 |
|  | single_MBEI_log2_alternative_group | Hu | 1.63 | 2.2E-06 | 2 | 1.33 |
|  | all_GCRMA_default_group | Hu | 1.5 | 1.1E-04 | 1.84 | 1.22 |
|  | all_MAS5_log2_default_group | Hu | 1.33 | 7.0E-03 | 1.63 | 1.08 |
|  | all_MBEI_log2_default_group | Hu | 1.38 | 1.9E-03 | 1.7 | 1.13 |
|  | all_GCRMA_alternative_group | Hu | 1.76 | 5.3E-08 | 2.16 | 1.44 |
|  | all_MAS5_log2_alternative_group | Hu | 1.49 | 1.3E-04 | 1.82 | 1.21 |
|  | all_MBEI_log2_alternative_group | Hu | 1.65 | 1.7E-06 | 2.02 | 1.34 |
| Unanimous classifier | |  |  |  |  |  |
|  | unanimous | Hu | 1.88 | 3.8E-05 | 2.54 | 1.39 |
| Random forest classifiers | |  |  |  |  |  |
|  | preprocessing ensemble (unanimous patients only) | Hu | 1.05 | 7.5E-01 | 1.4 | 0.79 |
|  | preprocessing ensemble (all patients) | Hu | 0.92 | 5.8E-01 | 1.24 | 0.68 |
|  | engineered variables (unanimous patients only) | Hu | 0.89 | 4.7E-01 | 1.21 | 0.66 |
|  | engineered variables (all patinets) | Hu | 1.62 | 4.6E-06 | 1.99 | 1.32 |
|  | preprocessing and engineered variables (all patients) | Hu | 1.62 | 4.4E-06 | 1.99 | 1.32 |
|  | Boruta algorithm selected features | Hu | 1.63 | 3.9E-06 | 2 | 1.32 |
| Preprocessing pipeline | |  |  |  |  |  |
|  | single_RMA_default_group | Sorensen | 1.23 | 4.6E-02 | 1.51 | 1 |
|  | single_MAS5_default_group | Sorensen | 1.26 | 2.8E-02 | 1.54 | 1.03 |
|  | single_MBEI_default_group | Sorensen | 1.64 | 2.9E-06 | 2.02 | 1.33 |
|  | single_GCRMA_default_group | Sorensen | 1.25 | 3.2E-02 | 1.53 | 1.02 |
|  | single_MAS5_log2_default_group | Sorensen | 1.26 | 2.8E-02 | 1.54 | 1.03 |
|  | single_MBEI_log2_default_group | Sorensen | 1.64 | 2.9E-06 | 2.02 | 1.33 |
|  | single_RMA_alternative_group | Sorensen | 1.34 | 4.9E-03 | 1.64 | 1.09 |
|  | single_MAS5_alternative_group | Sorensen | 1.18 | 1.1E-01 | 1.44 | 0.96 |
|  | single_MBEI_alternative_group | Sorensen | 1.51 | 8.2E-05 | 1.85 | 1.23 |
|  | single_GCRMA_alternative_group | Sorensen | 1.51 | 7.0E-05 | 1.86 | 1.23 |
|  | single_MAS5_log2_alternative_group | Sorensen | 1.18 | 1.1E-01 | 1.44 | 0.96 |
|  | single_MBEI_log2_alternative_group | Sorensen | 1.51 | 8.2E-05 | 1.85 | 1.23 |
|  | all_RMA_default_group | Sorensen | 1.26 | 2.5E-02 | 1.55 | 1.03 |
|  | all_MAS5_default_group | Sorensen | 1.3 | 1.2E-02 | 1.59 | 1.06 |
|  | all_MBEI_default_group | Sorensen | 1.34 | 5.3E-03 | 1.64 | 1.09 |
|  | all_GCRMA_default_group | Sorensen | 1.3 | 1.3E-02 | 1.59 | 1.06 |
|  | all_MAS5_log2_default_group | Sorensen | 1.3 | 1.2E-02 | 1.59 | 1.06 |
|  | all_MBEI_log2_default_group | Sorensen | 1.34 | 5.3E-03 | 1.64 | 1.09 |
|  | all_RMA_alternative_group | Sorensen | 1.4 | 1.3E-03 | 1.71 | 1.14 |
|  | all_MAS5_alternative_group | Sorensen | 1.22 | 5.8E-02 | 1.49 | 0.99 |
|  | all_MBEI_alternative_group | Sorensen | 1.42 | 7.1E-04 | 1.75 | 1.16 |
|  | all_GCRMA_alternative_group | Sorensen | 1.43 | 6.0E-04 | 1.76 | 1.17 |
|  | all_MAS5_log2_alternative_group | Sorensen | 1.22 | 5.8E-02 | 1.49 | 0.99 |
|  | all_MBEI_log2_alternative_group | Sorensen | 1.42 | 7.1E-04 | 1.75 | 1.16 |
| Unanimous classifier | |  |  |  |  |  |
|  | unanimous | Sorensen | 1.5 | 7.6E-03 | 2.02 | 1.11 |
| Random forest classifiers | |  |  |  |  |  |
|  | preprocessing ensemble (unanimous patients only) | Sorensen | 1.39 | 2.4E-02 | 1.85 | 1.04 |
|  | preprocessing ensemble (all patients) | Sorensen | 1.58 | 1.4E-05 | 1.93 | 1.28 |
|  | engineered variables (unanimous patients only) | Sorensen | 1.1 | 5.1E-01 | 1.48 | 0.82 |
|  | engineered variables (all patinets) | Sorensen | 1.17 | 1.3E-01 | 1.44 | 0.96 |
|  | preprocessing and engineered variables (all patients) | Sorensen | 1.29 | 1.4E-02 | 1.59 | 1.05 |
|  | Boruta algorithm selected features | Sorensen | 2.28 | 2.5E-16 | 2.78 | 1.87 |
