# Supplementary material for: Prediction of early breast cancer patient survival using ensembles of hypoxia signatures
Source: PLoS One. 2018 Sep 14;13(9):e0204123. doi: 10.1371/journal.pone.0204123 (PMC6138385; doi:10.1371/journal.pone.0204123)
Supplement: S6 Table — (DOCX) [file pone.0204123.s006.docx]

| Table S6 Hazard ratios and 95% confidence intervals obtained for each of the 24 preprocessing methods, the random forest classifiers evaluated, and the simple unanimous vote classifier, per signature (HG-U133 Plus 2.0 microarray platform). | | | | | | |
| --- | --- | --- | --- | --- | --- | --- |
| Classifier | | **Signature** | **HR** | ***P*-value** | **Upper 95% CI** | **Lower 95% CI** |
| Preprocessing pipeline | |  |  |  |  |  |
|  | single_RMA_default_group | Buffa | 1.83 | 9.3E-06 | 2.39 | 1.4 |
|  | single_MAS5_default_group | Buffa | 2.02 | 3.4E-07 | 2.64 | 1.54 |
|  | single_MBEI_default_group | Buffa | 1.81 | 1.4E-05 | 2.36 | 1.38 |
|  | single_GCRMA_default_group | Buffa | 1.93 | 1.5E-06 | 2.53 | 1.48 |
|  | single_MAS5_log2_default_group | Buffa | 2.02 | 3.4E-07 | 2.64 | 1.54 |
|  | single_MBEI_log2_default_group | Buffa | 1.81 | 1.4E-05 | 2.36 | 1.38 |
|  | single_RMA_alternative_group | Buffa | 1.81 | 1.1E-05 | 2.36 | 1.39 |
|  | single_MAS5_alternative_group | Buffa | 2.01 | 3.7E-07 | 2.63 | 1.54 |
|  | single_MBEI_alternative_group | Buffa | 1.89 | 2.4E-06 | 2.47 | 1.45 |
|  | single_GCRMA_alternative_group | Buffa | 1.92 | 1.6E-06 | 2.51 | 1.47 |
|  | single_MAS5_log2_alternative_group | Buffa | 2.01 | 3.7E-07 | 2.63 | 1.54 |
|  | single_MBEI_log2_alternative_group | Buffa | 1.89 | 2.4E-06 | 2.47 | 1.45 |
|  | all_RMA_default_group | Buffa | 2.02 | 1.2E-07 | 2.62 | 1.56 |
|  | all_MAS5_default_group | Buffa | 2.04 | 8.0E-08 | 2.65 | 1.57 |
|  | all_MAS5_log2_default_group | Buffa | 2.04 | 8.0E-08 | 2.65 | 1.57 |
|  | all_RMA_alternative_group | Buffa | 1.91 | 1.2E-06 | 2.48 | 1.47 |
|  | all_MAS5_alternative_group | Buffa | 1.81 | 8.2E-06 | 2.35 | 1.39 |
|  | all_MAS5_log2_alternative_group | Buffa | 1.81 | 8.2E-06 | 2.35 | 1.39 |
|  | all_MBEI_alternative_group | Buffa | 1.92 | 9.9E-07 | 2.49 | 1.48 |
|  | all_MBEI_log2_alternative_group | Buffa | 1.92 | 9.9E-07 | 2.49 | 1.48 |
|  | all_GCRMA_default_group | Buffa | 1.99 | 2.0E-07 | 2.59 | 1.54 |
|  | all_GCRMA_alternative_group | Buffa | 1.96 | 3.9E-07 | 2.55 | 1.51 |
|  | all_MBEI_default_group | Buffa | 2.01 | 1.6E-07 | 2.61 | 1.55 |
|  | all_MBEI_log2_default_group | Buffa | 2.01 | 1.6E-07 | 2.61 | 1.55 |
| Unanimous classifier | |  |  |  |  |  |
|  | unanimous | Buffa | 2.67 | 1.1E-09 | 3.67 | 1.95 |
| Random forest classifiers | |  |  |  |  |  |
|  | preprocessing and engineered variables (all patients) | Buffa | 1.99 | 2.7E-07 | 2.58 | 1.53 |
|  | preprocessing ensemble (all patients) | Buffa | 1.98 | 3.2E-07 | 2.57 | 1.52 |
|  | engineered variables (all patients) | Buffa | 1.98 | 3.3E-07 | 2.57 | 1.52 |
|  | Boruta algorithm selected features | Buffa | 2.01 | 1.4E-07 | 2.61 | 1.55 |
| Preprocessing pipeline | |  |  |  |  |  |
|  | single_RMA_default_group | Winter | 1.84 | 7.9E-06 | 2.4 | 1.41 |
|  | single_MAS5_default_group | Winter | 2.03 | 2.5E-07 | 2.66 | 1.55 |
|  | single_MBEI_default_group | Winter | 1.81 | 1.4E-05 | 2.36 | 1.38 |
|  | single_GCRMA_default_group | Winter | 1.89 | 3.2E-06 | 2.47 | 1.45 |
|  | single_MAS5_log2_default_group | Winter | 2.03 | 2.5E-07 | 2.66 | 1.55 |
|  | single_MBEI_log2_default_group | Winter | 1.81 | 1.4E-05 | 2.36 | 1.38 |
|  | single_RMA_alternative_group | Winter | 1.85 | 5.2E-06 | 2.42 | 1.42 |
|  | single_MAS5_alternative_group | Winter | 1.72 | 5.1E-05 | 2.24 | 1.32 |
|  | single_MBEI_alternative_group | Winter | 1.98 | 5.1E-07 | 2.58 | 1.52 |
|  | single_GCRMA_alternative_group | Winter | 1.85 | 5.7E-06 | 2.42 | 1.42 |
|  | single_MAS5_log2_alternative_group | Winter | 1.72 | 5.1E-05 | 2.24 | 1.32 |
|  | single_MBEI_log2_alternative_group | Winter | 1.98 | 5.1E-07 | 2.58 | 1.52 |
|  | all_RMA_default_group | Winter | 2.16 | 7.1E-09 | 2.8 | 1.66 |
|  | all_MAS5_default_group | Winter | 1.86 | 3.6E-06 | 2.41 | 1.43 |
|  | all_MAS5_log2_default_group | Winter | 1.86 | 3.6E-06 | 2.41 | 1.43 |
|  | all_RMA_alternative_group | Winter | 2.04 | 8.1E-08 | 2.65 | 1.57 |
|  | all_MAS5_alternative_group | Winter | 1.71 | 6.0E-05 | 2.22 | 1.31 |
|  | all_MAS5_log2_alternative_group | Winter | 1.71 | 6.0E-05 | 2.22 | 1.31 |
|  | all_MBEI_alternative_group | Winter | 1.79 | 1.2E-05 | 2.32 | 1.38 |
|  | all_MBEI_log2_alternative_group | Winter | 1.79 | 1.2E-05 | 2.32 | 1.38 |
|  | all_MBEI_default_group | Winter | 2.16 | 8.5E-09 | 2.81 | 1.66 |
|  | all_MBEI_log2_default_group | Winter | 2.16 | 8.5E-09 | 2.81 | 1.66 |
|  | all_GCRMA_default_group | Winter | 2.15 | 8.3E-09 | 2.79 | 1.66 |
|  | all_GCRMA_alternative_group | Winter | 1.74 | 3.2E-05 | 2.26 | 1.34 |
| Unanimous classifier | |  |  |  |  |  |
|  | unanimous | Winter | 2.53 | 1.9E-08 | 3.5 | 1.83 |
| Random forest classifiers | |  |  |  |  |  |
|  | preprocessing and engineered variables (all patients) | Winter | 1.83 | 5.1E-06 | 2.38 | 1.41 |
|  | preprocessing ensemble (all patients) | Winter | 1.85 | 3.7E-06 | 2.4 | 1.43 |
|  | engineered variables (all patients) | Winter | 1.85 | 4.0E-06 | 2.4 | 1.42 |
|  | Boruta algorithm selected features | Winter | 2.52 | 4.0E-12 | 3.28 | 1.94 |
| Preprocessing pipeline | |  |  |  |  |  |
|  | single_RMA_default_group | Hu | 1.71 | 7.6E-05 | 2.22 | 1.31 |
|  | single_MAS5_default_group | Hu | 1.71 | 7.0E-05 | 2.23 | 1.31 |
|  | single_MBEI_default_group | Hu | 1.75 | 3.3E-05 | 2.29 | 1.35 |
|  | single_GCRMA_default_group | Hu | 1.65 | 2.0E-04 | 2.15 | 1.27 |
|  | single_MAS5_log2_default_group | Hu | 1.71 | 7.0E-05 | 2.23 | 1.31 |
|  | single_MBEI_log2_default_group | Hu | 1.75 | 3.3E-05 | 2.29 | 1.35 |
|  | single_RMA_alternative_group | Hu | 1.89 | 1.9E-06 | 2.45 | 1.45 |
|  | single_MAS5_alternative_group | Hu | 1.8 | 9.2E-06 | 2.34 | 1.39 |
|  | single_MBEI_alternative_group | Hu | 1.84 | 4.6E-06 | 2.39 | 1.42 |
|  | single_GCRMA_alternative_group | Hu | 1.9 | 1.5E-06 | 2.46 | 1.46 |
|  | single_MAS5_log2_alternative_group | Hu | 1.8 | 9.2E-06 | 2.34 | 1.39 |
|  | single_MBEI_log2_alternative_group | Hu | 1.84 | 4.6E-06 | 2.39 | 1.42 |
|  | all_RMA_default_group | Hu | 2.12 | 2.8E-08 | 2.76 | 1.63 |
|  | all_MAS5_default_group | Hu | 2.01 | 2.9E-07 | 2.62 | 1.54 |
|  | all_MAS5_log2_default_group | Hu | 2.01 | 2.9E-07 | 2.62 | 1.54 |
|  | all_RMA_alternative_group | Hu | 2.19 | 3.7E-09 | 2.85 | 1.69 |
|  | all_MAS5_alternative_group | Hu | 2.27 | 1.0E-09 | 2.96 | 1.75 |
|  | all_MAS5_log2_alternative_group | Hu | 2.27 | 1.0E-09 | 2.96 | 1.75 |
|  | all_MBEI_alternative_group | Hu | 2.39 | 7.4E-11 | 3.11 | 1.84 |
|  | all_MBEI_log2_alternative_group | Hu | 2.39 | 7.4E-11 | 3.11 | 1.84 |
|  | all_GCRMA_default_group | Hu | 1.89 | 2.0E-06 | 2.47 | 1.46 |
|  | all_GCRMA_alternative_group | Hu | 2 | 1.9E-07 | 2.59 | 1.54 |
|  | all_MBEI_default_group | Hu | 2.19 | 9.9E-09 | 2.86 | 1.67 |
|  | all_MBEI_log2_default_group | Hu | 2.19 | 9.9E-09 | 2.86 | 1.67 |
| Unanimous classifier | |  |  |  |  |  |
|  | unanimous | Hu | 2.5 | 4.9E-08 | 3.47 | 1.8 |
| Random forest classifiers | |  |  |  |  |  |
|  | preprocessing and engineered variables (all patients) | Hu | 2.21 | 6.2E-09 | 2.88 | 1.69 |
|  | preprocessing ensemble (all patients) | Hu | 2.12 | 3.1E-08 | 2.77 | 1.63 |
|  | engineered variables (all patients) | Hu | 2.35 | 3.3E-10 | 3.06 | 1.8 |
|  | Boruta algorithm selected features | Hu | 2.25 | 3.3E-09 | 2.94 | 1.72 |
| Preprocessing pipeline | |  |  |  |  |  |
|  | single_RMA_default_group | Sorensen | 1.47 | 4.0E-03 | 1.91 | 1.13 |
|  | single_MAS5_default_group | Sorensen | 1.21 | 1.5E-01 | 1.57 | 0.93 |
|  | single_MBEI_default_group | Sorensen | 1.42 | 9.0E-03 | 1.84 | 1.09 |
|  | single_GCRMA_default_group | Sorensen | 1.42 | 9.1E-03 | 1.84 | 1.09 |
|  | single_MAS5_log2_default_group | Sorensen | 1.21 | 1.5E-01 | 1.57 | 0.93 |
|  | single_MBEI_log2_default_group | Sorensen | 1.42 | 9.0E-03 | 1.84 | 1.09 |
|  | single_RMA_alternative_group | Sorensen | 1.39 | 1.3E-02 | 1.81 | 1.07 |
|  | single_MAS5_alternative_group | Sorensen | 1.18 | 2.1E-01 | 1.53 | 0.91 |
|  | single_MBEI_alternative_group | Sorensen | 1.22 | 1.4E-01 | 1.58 | 0.94 |
|  | single_GCRMA_alternative_group | Sorensen | 1.3 | 4.9E-02 | 1.69 | 1 |
|  | single_MAS5_log2_alternative_group | Sorensen | 1.18 | 2.1E-01 | 1.53 | 0.91 |
|  | single_MBEI_log2_alternative_group | Sorensen | 1.22 | 1.4E-01 | 1.58 | 0.94 |
|  | all_RMA_default_group | Sorensen | 1.65 | 1.6E-04 | 2.14 | 1.27 |
|  | all_MAS5_default_group | Sorensen | 1.56 | 8.0E-04 | 2.03 | 1.2 |
|  | all_MAS5_log2_default_group | Sorensen | 1.56 | 8.0E-04 | 2.03 | 1.2 |
|  | all_RMA_alternative_group | Sorensen | 1.51 | 2.2E-03 | 1.96 | 1.16 |
|  | all_MAS5_alternative_group | Sorensen | 1.43 | 7.2E-03 | 1.86 | 1.1 |
|  | all_MAS5_log2_alternative_group | Sorensen | 1.43 | 7.2E-03 | 1.86 | 1.1 |
|  | all_MBEI_alternative_group | Sorensen | 1.34 | 3.0E-02 | 1.75 | 1.03 |
|  | all_MBEI_log2_alternative_group | Sorensen | 1.34 | 3.0E-02 | 1.75 | 1.03 |
|  | all_GCRMA_default_group | Sorensen | 1.68 | 9.4E-05 | 2.18 | 1.29 |
|  | all_GCRMA_alternative_group | Sorensen | 1.37 | 1.7E-02 | 1.78 | 1.06 |
|  | all_MBEI_default_group | Sorensen | 1.87 | 2.6E-06 | 2.43 | 1.44 |
|  | all_MBEI_log2_default_group | Sorensen | 1.87 | 2.6E-06 | 2.43 | 1.44 |
| Unanimous classifier | |  |  |  |  |  |
|  | unanimous | Sorensen | 1.81 | 8.8E-04 | 2.57 | 1.28 |
| Random forest classifiers | |  |  |  |  |  |
|  | preprocessing and engineered variables (all patients) | Sorensen | 1.72 | 4.3E-05 | 2.24 | 1.33 |
|  | preprocessing ensemble (all patients) | Sorensen | 1.59 | 5.4E-04 | 2.06 | 1.22 |
|  | engineered variables (all patients) | Sorensen | 1.7 | 7.5E-05 | 2.2 | 1.31 |
|  | Boruta algorithm selected features | Sorensen | 1.87 | 2.6E-06 | 2.42 | 1.44 |
