# Supplementary material for: Prediction of early breast cancer patient survival using ensembles of hypoxia signatures
Source: PLoS One. 2018 Sep 14;13(9):e0204123. doi: 10.1371/journal.pone.0204123 (PMC6138385; doi:10.1371/journal.pone.0204123)
Supplement: S7 Table — (DOCX) [file pone.0204123.s007.docx]

| Table S7 Frequency of top ranked genes selected from each of the preprocessing pipelines by univariate Cox proportional hazard models. | | | | | | | |
| --- | --- | --- | --- | --- | --- | --- | --- |
| Gene | **Frequency** | **Gene** | **Frequency** | **Gene** | **Frequency** | **Gene** | **Frequency** |
| AURKA | 12 | STK26 | 10 | SPC25 | 4 | CCT2 | 2 |
| TOP2A | 12 | CX3CR1 | 9 | DPM1 | 4 | MMP1 | 2 |
| RRM2 | 12 | RFC4 | 9 | FBLN5 | 4 | NQO1 | 2 |
| CDK1 | 12 | CENPE | 8 | FBXO5 | 4 | PGAM1 | 2 |
| CENPF | 12 | CENPM | 8 | PDGFD | 4 | PWP2 | 2 |
| FANCI | 12 | CENPN | 8 | PGR | 4 | TUBB4B | 2 |
| CCNA2 | 12 | HJURP | 8 | PLK4 | 4 | AQP1 | 2 |
| CXCL12 | 12 | KIF11 | 8 | SMARCA2 | 4 | CDCA8 | 2 |
| ABCC5 | 12 | MCM10 | 8 | ALG3 | 4 | NMU | 2 |
| ASPM | 12 | NEK2 | 8 | ENPP2 | 4 | RECQL4 | 2 |
| CCNB2 | 12 | OIP5 | 8 | JCHAIN | 4 | ZDHHC13 | 2 |
| CCNE2 | 12 | PGK1 | 8 | SMYD2 | 4 | GINS3 | 2 |
| CDKN3 | 12 | STIL | 8 | TSPAN7 | 4 | CD1C | 2 |
| CENPU | 12 | TTK | 8 | ITM2A | 4 | CECR5 | 2 |
| DLGAP5 | 12 | TUBA1B | 8 | STMN1 | 4 | DIRAS3 | 2 |
| DTL | 12 | MCM2 | 8 | KIAA0101 | 4 | ENTPD1 | 2 |
| E2F8 | 12 | EZH2 | 8 | EMCN | 3 | EPB41L2 | 2 |
| ECT2 | 12 | SPARCL1 | 8 | FBXW4 | 3 | HN1 | 2 |
| ESPL1 | 12 | TACC3 | 8 | HLA-E | 3 | IGLV2-14 | 2 |
| FOXM1 | 12 | CDC6 | 7 | TROAP | 3 | KIAA0430 | 2 |
| GINS1 | 12 | APOBEC3B | 7 | TUBA1C | 3 | SPATA6 | 2 |
| KIF14 | 12 | EXO1 | 6 | CDCA3 | 3 | TGFB3 | 2 |
| KIF20A | 12 | FEN1 | 6 | CKS2 | 3 | ZWINT | 2 |
| KIF2C | 12 | MFAP4 | 6 | CSE1L | 3 | ARID5B | 2 |
| KIF4A | 12 | NTRK2 | 6 | CTSV | 3 | ARPC5L | 2 |
| MAD2L1 | 12 | SQLE | 6 | HSP90AA1 | 3 | CH25H | 2 |
| MELK | 12 | TPX2 | 6 | CCNE1 | 3 | GINS2 | 2 |
| NCAPG | 12 | ZFP36L2 | 6 | LINC00341 | 3 | IDI1 | 2 |
| NUSAP1 | 12 | DONSON | 6 | NUP155 | 3 | MPC2 | 2 |
| PRC1 | 12 | EPHX2 | 6 | STARD13 | 3 | NDUFS6 | 2 |
| RACGAP1 | 12 | LAMA2 | 6 | GTPBP4 | 3 | SEPP1 | 2 |
| RAD51AP1 | 12 | MCM6 | 6 | KCTD12 | 3 | SRSF5 | 2 |
| SPAG5 | 12 | PTTG1 | 6 | MIS18A | 3 | STAT5B | 2 |
| TRIP13 | 12 | TXNIP | 6 | LRIG1 | 3 | CD200 | 1 |
| UBE2C | 12 | AURKB | 6 | N4BP2L1 | 3 | CENPI | 1 |
| BIRC5 | 11 | CENPA | 6 | CDC25A | 2 | CREBL2 | 1 |
| BUB1 | 11 | ORC6 | 6 | FAM64A | 2 | DNAJC9 | 1 |
| GTSE1 | 11 | CDC45 | 5 | HMMR | 2 | DSN1 | 1 |
| MYBL2 | 11 | DIXDC1 | 5 | HPSE | 2 | E2F1 | 1 |
| BUB1B | 11 | ADGRG1 | 5 | INPP1 | 2 | JADE2 | 1 |
| CDC20 | 11 | DSCC1 | 5 | KIF15 | 2 | KPNA2 | 1 |
| CMC2 | 11 | NCAPH | 5 | RAI2 | 2 | MAGOHB | 1 |
| SIK3 | 11 | PSMD2 | 5 | TIMELESS | 2 | MTUS1 | 1 |
| CEP55 | 10 | TIMM17A | 5 | IGHD | 2 | SKP2 | 1 |
| IGF1 | 10 | C3 | 5 | PSMA7 | 2 | CYBRD1 | 1 |
| KIF18A | 10 | TK1 | 5 | SLC19A1 | 2 | DNMT3B | 1 |
| KIF23 | 10 | SETBP1 | 5 | UBE2A | 2 | FAS | 1 |
| MKI67 | 10 | FRZB | 5 | VPS41 | 2 | OMD | 1 |
| NDC80 | 10 | KIF18B | 4 | ANP32E | 2 | SDS | 1 |
| SHCBP1 | 10 | RAD51 | 4 | ARHGEF12 | 2 | SLIT2 | 1 |
