# Supplementary material for: Prediction of early breast cancer patient survival using ensembles of hypoxia signatures
Source: PLoS One. 2018 Sep 14;13(9):e0204123. doi: 10.1371/journal.pone.0204123 (PMC6138385; doi:10.1371/journal.pone.0204123)
Supplement: S1 Fig — (A) Forest plot of log2 hazard ratios with 95% confidence intervals obtained for each of the 24 preprocessing (PP) methods, the random forest classifiers evaluated, and the simple unanimous vote classifier (total number of votes for poor prognosis either 0 or 24). The forest plot is ordered as decreasing hazard ratio. The dotted line represents a hazard ratio of 1. The blue hazard ratio with its 95% confidence interval represents the hazard ratio for the simple unanimous vote classifier. (B) Bar plot of accuracy obtained for each of the 24 preprocessing methods, the random forest classifiers evaluated, and the simple unanimous vote classifier. The bars are ordered by preprocessing pipelines, the unanimous classifier, and the best performing random forest classifier, from left to right. (DOCX) [file pone.0204123.s008.docx]

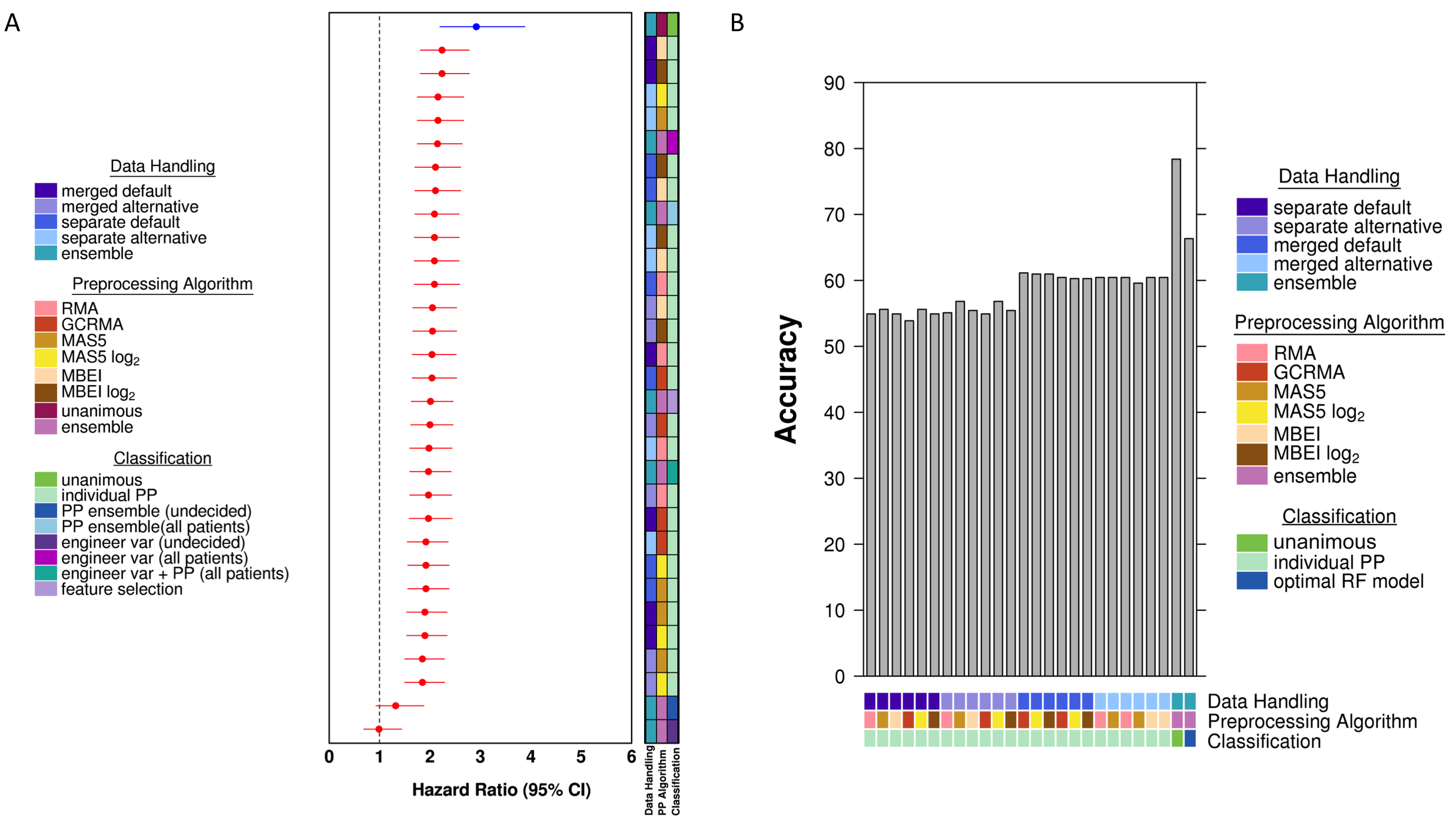


**Figure S1** Hazard ratio forest plot and accuracy for Buffa metagene signature using the HG-U133A microarray platform. (A) Forest plot of log_2_ hazard ratios with 95% confidence intervals obtained for each of the 24 preprocessing methods, the random forest classifiers evaluated, and the simple unanimous vote classifier (total number of votes for poor prognosis either 0 or 24). The forest plot is ordered as decreasing hazard ratio. The dotted line represents a hazard ratio of 1. The blue hazard ratio with its 95% confidence interval represents the hazard ratio for the simple unanimous vote classifier. (B) Bar plot of accuracy obtained for each of the 24 preprocessing methods, the random forest classifiers evaluated, and the simple unanimous vote classifier. The bars are ordered by preprocessing pipelines, the unanimous classifier, and the best performing random forest classifier, from left to right.
