# Supplementary material for: Prediction of early breast cancer patient survival using ensembles of hypoxia signatures
Source: PLoS One. 2018 Sep 14;13(9):e0204123. doi: 10.1371/journal.pone.0204123 (PMC6138385; doi:10.1371/journal.pone.0204123)
Supplement: S12 Fig — (A) Prognostic ability of signature in patients with unanimous ensemble agreement across preprocessing pipelines. (B) Prognostic ability of signature classification using the best performing preprocessing pipeline. (C) Prognostic ability of signature classification using the best performing random forest-based ensemble of preprocessing pipelines. Hazard ratios and p-values are from Cox proportional hazard ratio modeling. (DOCX) [file pone.0204123.s019.docx]

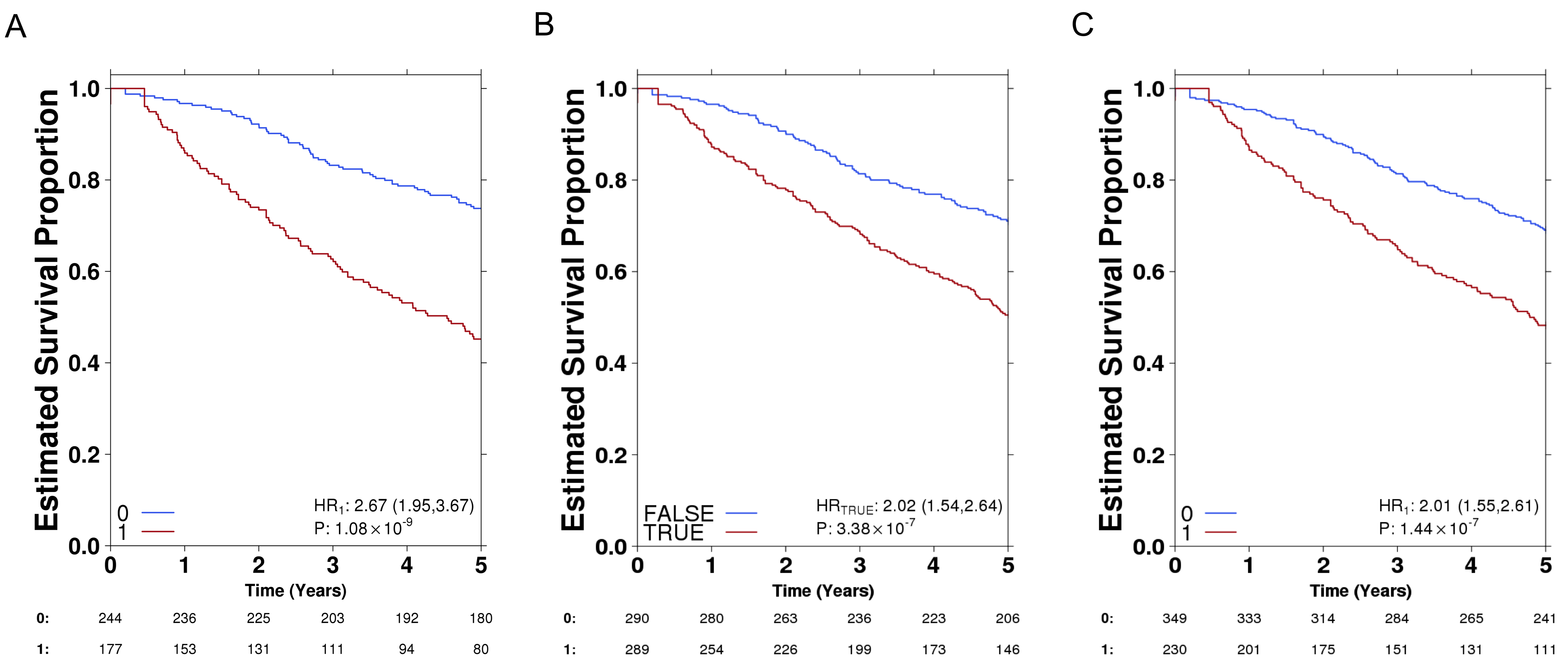


**Figure S12** Kaplan-Meier survival curves evaluating the prognostic ability of the Buffa metagene signature using HG-U133 Plus 2.0 microarray platform. (A) Prognostic ability of signature in patients with unanimous ensemble agreement across preprocessing pipelines. (B) Prognostic ability of signature classification using the best performing preprocessing pipeline. (C) Prognostic ability of signature classification using the best performing random forest-based ensemble of preprocessing pipelines. Hazard ratios and p-values are from Cox proportional hazard ratio modeling.
