# Supplementary material for: Prediction of early breast cancer patient survival using ensembles of hypoxia signatures
Source: PLoS One. 2018 Sep 14;13(9):e0204123. doi: 10.1371/journal.pone.0204123 (PMC6138385; doi:10.1371/journal.pone.0204123)
Supplement: S16 Fig — (A) Similar to Fig 5, hazard ratios with 95% confidence intervals of novel signature classifiers ordered by decreasing hazard ratios. Dotted line represents a hazard ratio of 1. (B) Class predictions of biochemical recurrence from each of the classifiers and the ensemble. Each row is a classifier and are ordered by decreasing hazard ratios, while each column is a patient and are ordered by agreement across the 13 classifiers. The true class for each patient are indicated by the bar on top. A predicted class label matching the true label is blue for true positive, and purple for true negative, while white indicates an incorrect label. (DOCX) [file pone.0204123.s023.docx]

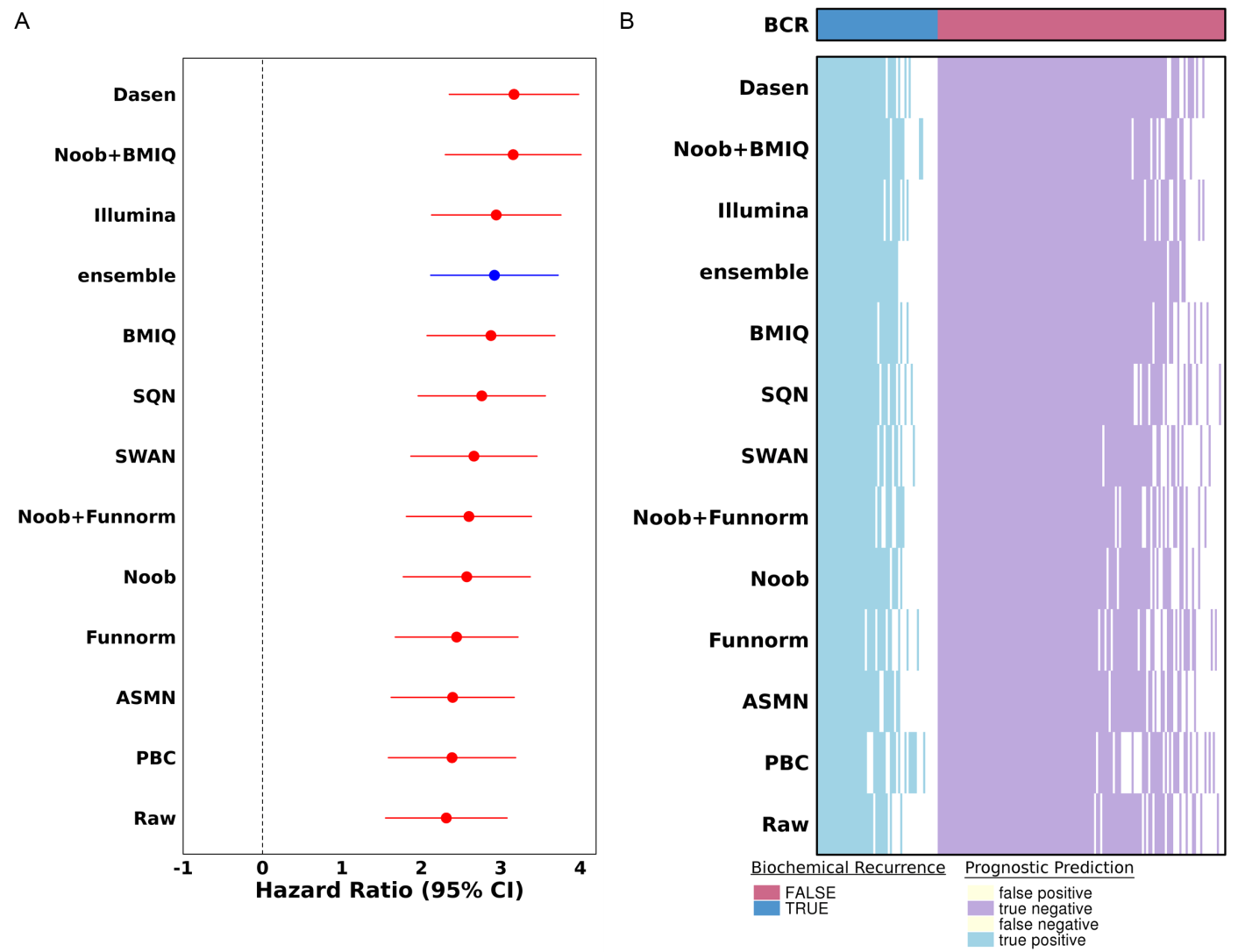


**Figure S16** Hazard ratios of classifier performance and their predictions of biochemical recurrence in different methylation preprocessing methods for intermediate-risk prostate cancer patients. (A) Similar to figure 5, hazard ratios with 95% confidence intervals of novel signature classifiers ordered by decreasing hazard ratios. Dotted line represents a hazard ratio of 1. (B) Class predictions of biochemical recurrence from each of the classifiers and the ensemble. Each row is a classifier and are ordered by decreasing hazard ratios, while each column is a patient and are ordered by agreement across the 13 classifiers. The true class for each patient are indicated by the bar on top. A predicted class label matching the true label is blue for true positive, and purple for true negative, while white indicates an incorrect label.
